# Improving wear resistance of plasma-sprayed calcia and magnesia-stabilized zirconia mixed coating: roles of phase stability and microstructure

**DOI:** 10.1038/s41598-020-78088-6

**Published:** 2020-12-11

**Authors:** Mohamed Abd-Elsattar Hafez, Sameh Ahmed Akila, Mohamed Atta Khedr, Ali Saeid Khalil

**Affiliations:** 1 Department of Laser Sciences and Interactions, National Institute of Laser Enhanced Sciences, Cairo University, Giza, 12613 Egypt; 2 Metallurgy and Mining Department, Tabbin Institute for Metallurgical Studies, POB 109, Helwan, Cairo, Egypt

**Keywords:** Materials science, Physics

## Abstract

The phase stability and microstructure of ZrO_2_–5CaO and ZrO_2_–24MgO mixed coating (wt%) by air plasma spraying on 304 stainless steel substrates were investigated. A Ni–5Al (wt%) metallic bond coating was firstly sprayed between the substrate and the ceramic top layer. The results were compared with the individual coatings of ZrO_2_–5CaO and ZrO_2_–24MgO for a better understanding of the correlation between their microstructures and mechanical properties. Mixed zirconia coating was found to have a mixture of cubic and tetragonal phases that stabilized under different plasma spray conditions. Microscopic observations and elemental composition analysis of as-sprayed mixed coating showed that modified ceramic-matrix grains had been formed. Microsized ZrO_2_–5CaO particles were embedded in the matrix grain creating an intragranular microstructure. Results indicated that ceramic-matrix grains provided a diffusion barrier for the growth of oxides induced stress near and onto the bond layer that reduced cracks, thereby overcoming the top delamination of the ceramic coating. Moreover, disparity in wear resistance and microhardness behavior of the coatings was influenced by initial feedstock powder and matrix microstructures. Improvement in the wear resistance of the mixed zirconia coating was attributed to a decrease in oxide content, which resulted in an increase in intersplat cohesive strength.

## Introduction

Next generation of thermal barrier coatings (TBCs) is currently the subject of extensive research in order to achieve more efficient performance over longer lifetimes^1–4^. Ceramic TBCs are used in gas turbine engines to shield metallic parts surfaces from intense heat exposure and subsequent degradation. However, the increasing demands for higher operating temperatures in aeroengines and power generation applications requires improvement of resistant coatings against oxidation, hot corrosion, and erosion wear^5–8^. Durable coatings largely depend on their mechanical properties and their adhesion to metallic substrates. Thus the main motivation to improve TBCs is to develop adequate coating systems to provide surface protection at high temperatures for long-term efficiency and durability.

TBCs are widely deposited by atmospheric plasma spraying (APS), electron beam physical vapor deposition (EBPVD), and high velocity oxy-fuel (HVOF). Deposition species and growth mechanisms by the APS, EBPVD, and HVOF methods have profound effects on the microstructures and properties of the processed coatings^9–13^. The TBCs consist of an outer ceramic top coating and an inner metallic bond coating deposited directly on the substrate. The advantages of using the APS approach include a high deposition rate, a wide range of coating thickness, and low processing costs. In this case, the structure of thermal sprayed coatings is lamellar, consisting of splats that are molten particles flattened by impact with the surface and then rapidly quenched^14^. The rapid particle cooling rate and lamellar splat structure are two features that distinguish thermal spray coatings. The differences in coating microstructure of the APS, which is porous and lamellar, and EBPVD, which is columnar, cause differences in the TBCs properties such as interlamellar or gradient porosity^15^. The quality of TBCs depends on spray process variables such as feedstock materials, temperature control, and coating buildup. However, defects that degrade the mechanical properties are commonly introduced by decreasing the wear resistance of the TBCs coatings and increasing the risk of corrosive and heat-resistant failure. Coating strength is affected by bond interfaces, pores, interlamellar oxide boundaries, phases, and unmelted particles within the deposit. Therefore, the need to control coatings defects and overcome the problems associated with rapid thermal and cooling processes are essential for producing highest bond strength and reliable wear resistance coatings.

Ceramic materials for elevated temperature resistance applications should be selected to attain optimal microstructure and hardness. Zirconia (ZrO_2_)-based TBCs deposited by APS have received considerable attention because of its physical properties such as low thermal conductivity, high melting point, and high strength. Zirconia ceramics as wear resistance material have been considered for engineering applications, which strongly depend on both crystal structure and phase transformations^16–18^. Pure ZrO_2_ has three polymorphs which exist in monoclinic, tetragonal, and cubic phases^19^. However, phase transformation of ZrO_2_ from the tetragonal to monoclinic phases is associated by a large shear strain and volume increase of ~ 3 to 5% upon cooling from high temperatures leading to residual stresses^19–21^. Reversible transformation of the monoclinic to the tetragonal phase in the temperature range of 950–1170 °C caused microcracks that spread and turned into macro cracks^21,22^. These phase transformations generally result in cracking and contributes to the failure of the TBC system. Metal oxides are added to ZrO_2_, such as CaO (5 wt%), MgO (15–24 wt%), or Y_2_O_3_ (6–12 wt%) to stabilize the tetragonal and/or cubic phases at the ambient temperature condition^19,23^. The phase transformation is affected by various conditions, such as cooling rate and coating microstructure. Fast cooling rate, such as air cooling and water quenching, of ZrO_2_–8Y_2_O_3_ coating by APS showed less amount of monoclinic phase^24^. Occurrence of high temperature oxidation at the bond coating induces stress, which influence the lifetime of TBC. At elevated temperatures, oxygen transfers through the top coating towards the bond coating through intrinsic pores and microcracks of the coating to form an oxidized scale on the bond coating surface, which is termed thermally grown oxide (TGO). The TGO thickness can increase during high temperature oxidation process which causes stress and consequently spallation of coating at the interface of bond coat/ceramic top coat. One approach to overcome such mode of failure was using zirconia/Al_2_O_3_ composite coating to diminish the rate of TGO growth^25^. Using finite element model, it was found that TBC failure occurs through crack propagation conducted by the bond coat oxidation and the residual stresses that resulting from the expansion of the TGO^26^. Mechanical properties of TBCs are dependent principally on the resultant residual stresses that may relax to form cracks or the tendency to concentrate near the top coat/bond coat interface, which can cause the coating to spall from the substrate^27–29^.

The use of well established commercial ZrO_2_–CaO and ZrO_2_–MgO ceramic powders is characterized by the excellent ability to act as abrasion resistance and thermal load prevention coatings. Although ZrO_2_–Y_2_O_3_ is the most widely studied and applied ceramic layer material for TBC, it is more expensive. However, the CaO and MgO-stabilized zirconia have the merit of cost-effective coatings. Calcia addition is preferred over other oxides due to stability of cubic phase at all temperatures, whereas the magnesia or yttria-stabilization reverts to the monoclinic structure at low temperatures^30–32^. Therefore, calcia partially stabilizes the zirconia, which helps to reduce phase transformations as the coating passes through critical temperature regions. Furthermore, ZrO_2_–MgO has low thermal conductivity (1.0–1.5 W/mK), fairly comparable to ZrO_2_–Y_2_O_3_ which suggests that it has an enormous potential to be used as ceramic top coat material for suitable applications^33–35^. The desirability of diminishing surface degradation and disruption in the coating layers is based on phase stability and improved microstructure of the TBCs. The number of materials that can be used as TBCs is limited, therefore development of TBCs should be shifted towards different material combinations and deposition processes^36^. A multilayer system was employed for improvement of the thermal shock life of TBCs instead of using single material^37^. It has been reported that a dispersion of metastable tetragonal zirconia in cubic zirconia, a mixed phases, can be achieved and that gives rise to a more strengthening mechanism than partially-stabilized zirconia^21^.

In the present work, phase stability and microstructure of ZrO_2_–5CaO and ZrO_2_–24MgO mixed zirconia coatings by plasma spraying were investigated. Establishing optimal spraying conditions to benefit from the distinct properties of calcium and magnesia-stabilized zirconia could have a significant effect on TBCs. This study gives clearer insight into microstructure development of the plasma-sprayed mixed powders of calcia and magnesia-stabilized zirconia on Ni–5Al/304 stainless steel substrate and its impact on wear resistance and microhardness of the resulting coatings. Three coating systems of ZrO_2_–5CaO, ZrO_2_–24MgO, and their mixture were studied and discussed in order to better understand the effects of feedstock powder in different metals. In addition, the roles of phase stability and microstructure to improve the wear resistance of the top ceramic coatings are discussed.

## Materials and methods

### Substrate preparation and coating materials

The substrate used for the coatings was 304 stainless steel in the form of 20 mm diameter discs and 6–8 mm thick. Prior to the spraying of the calcia and magnesia stabilized zirconia coatings, the substrate was cleaned by degreasing its surface and then roughening by grit blasting with Corundum-500 (grain size 400:800 μm) at a pressure of 4 bar to obtain better adhesion. Grit blasting was carried out using Plasma-Technik AG, Switzerland. The standoff distance in shot blasting was kept between 100–150 mm and impact angle at 75°. The grit blasted substrates were subsequently cleaned with pressurized air in order to remove the remaining surface contamination. The chemical composition of the stainless steel substrate is given in Table 1. The initial powder materials were supplied from Sulzer Metco, USA. The bond layer was used to protect the stainless steel substrate from oxidation and corrosion at high temperatures has to be applied first to the substrate. The powder used for the bond coating was Nickel Aluminum (Ni–5 wt% Al), which is symbolize as Ni–5Al. The powders used for the top coatings were calcia-stabilized zirconium oxide (ZrO_2_–5 wt% CaO) and magnesia-stabilized zirconium oxide (ZrO_2_–24 wt% MgO), which are designated as CSZ and MSZ, respectively. Equal measured weights of the two CSZ and MSZ powders (measured using a sensitive digital balance with an accuracy of 10^–4^ g) were thoroughly mixed in 50:50 prior to spraying using a factory-based powder mixer. This mixture was sprayed as a top coating, designated here as CSZ-MSZ. Table 2 lists the starting powders characteristics.

**Table 1:** Chemical composition of the 304 stainless steel substrate.

Element	Cr	Ni	Mn Max	Si Max	P Max	S Max	C	Others
Weight %	17–19.5	8–10.5	2.0	1.0	0.045	0.030	≤ 0.07	N ≤ 0.11

**Table 2:** Characteristics of powders.

Powder	Ni–5 wt% Al	ZrO_2_–5 wt% CaO	ZrO_2_–24 wt% MgO
Particle shape	Spheroidal	Angular and blocky	Spheroidal
Particle size range (μm)	− 90 + 45	− 75 + 30	− 90 + 11
Manufacture	Mechanically Clad	Fused and crushed	Fused and crushed
Product reference	Metco 450NS	Metco 201B-NS	Metco 210NS-1

### Air plasma spraying system

The bond and ceramic top coatings were deposited on the substrate by the APS using a Plasma-Technik AG-Switzerland and robotic-plasma torch type F4-MB. The APS system consists of chamber of the plasma spray, the powder feeder system, the gas feeding system, and the control unit. The plasma spray chamber contains the plasma torch and rotatable table, which has the substrate holder and air-cooled pipeline. Different numbers of plasma torch scans were performed over the substrate to achieve the required top coating thickness. The thickness of the bond layer was prepared at approximately 50 μm. The experimental errors in measuring the coating thickness were estimated to be ± 14%. The top coatings of the samples were sprayed at selected plasma input currents of 600, 650, and 700 A. The spraying parameters used for the Ni-5Al bond coating and ceramic top coatings are summarized in Table 3. Coated samples of the CSZ, MSZ, and CSZ-MSZ powders with different plasma spraying currents were designated as ZC, ZM, and Mix, respectively. Table 4 lists the designated top-coated samples with the applied plasma current. The bond coat of Ni–5Al was first applied, followed by the top coat.

**Table 3:** Operating parameters for plasma spraying.

Parameters	Bond coat	Top ceramic coat
Primary gas rate (Argon) (l/min)	50	37.5
Secondary gas rate (Hydrogen) (l/min)	10	19
Current (A)	500	600, 650, 700
Voltage (V)	69	72
Nozzle diameter (mm)	6	6
Powder carrier gas rate (Argon) (l/min)	3.4	3.4
Powder feed rate (g/min)	60–68	38–40
Spray distance (mm)	140–178	102–125
Cooling	Air	Air

**Table 4:** Symbols of top coated samples with plasma current.

Current (A)	ZrO_2_–5CaO (CSZ)	ZrO_2_–24MgO (MSZ)	Mixed (CSZ-MSZ)
600	ZC1	ZM1	Mix1
650	ZC2	ZM2	Mix2
700	ZC3	ZM3	Mix3

### Powders and coatings characterization

The microstructure, phase composition, microhardness, and wear resistance of the as-sprayed coatings were characterized. Top surfaces and cross-sections of the sprayed coatings were prepared before examination. Samples were sectioned, mounted, grind, and polished to investigate the as-sprayed coatings and interfaces. The polished cross sections and top surfaces of the coatings were then analyzed using X-ray diffraction (XRD) analysis. This was used for phase identification of the starting powders and the as-sprayed coatings. XRD diffractometer model PANalytical system was utilized to analyze the powders (working conditions of 45 kV and 30 mA) and the as-sprayed coating samples (working conditions 45 kV and 40 mA). Measurements were carried out using CuKα radiation source of wavelength λ = 1.54 Å. The obtained XRD patterns were compared with d-spacing values taken from JCPDS cards to verify the diffracted peak indices for ZrO_2_, CaO, MgO, and NiAl. The patterns of XRD were obtained in the range of 25° ≤ 2θ ≤ 80°. Scanning electron microscopy (SEM) was used to characterize the microstructures of the starting powders, the top coating surfaces, and cross-sectioned samples. Chemical composition profiles in as-sprayed coated samples were analyzed by energy dispersive X-ray spectroscopy (EDX).

Rockwell hardness of top coating surfaces was measured by Digirock-RB tester (BMS BULUT Makina) under a 500 g load. Microhardness of the coatings were conducted using a Vickers micro-indenter (LECO LM70-Germany) at an applied load of 50 g (HV_0.05_) and a dwell time of 10 s. Indentation scans for the top coating, bond coating, and substrate were performed on polished cross-sections. The spacing between indentations (~ 630 μm) was made so that the separation distances are of at least three times the diagonals of the indentation in order to avoid stress-field effects from nearby indents. The abrasive wear resistance of the zirconia coatings was performed at room temperature without lubricant. Each sample was fitted to a special home-built holder into a pin on ring type TNO-TRIBO wear testing machine. A stainless steel ring (disc) rotates in contact with sample surface at rotating speed of 150 rpm (800 mm/s) under 4 N/cm^2^ load for duration of 180 s. The wear rate was determined by measuring the weight loss by weighing each sample before and after the test using a sensitive digital balance with an accuracy of 10^–4^ g.

## Results

### Phase composition analyses

**Figure 1:**
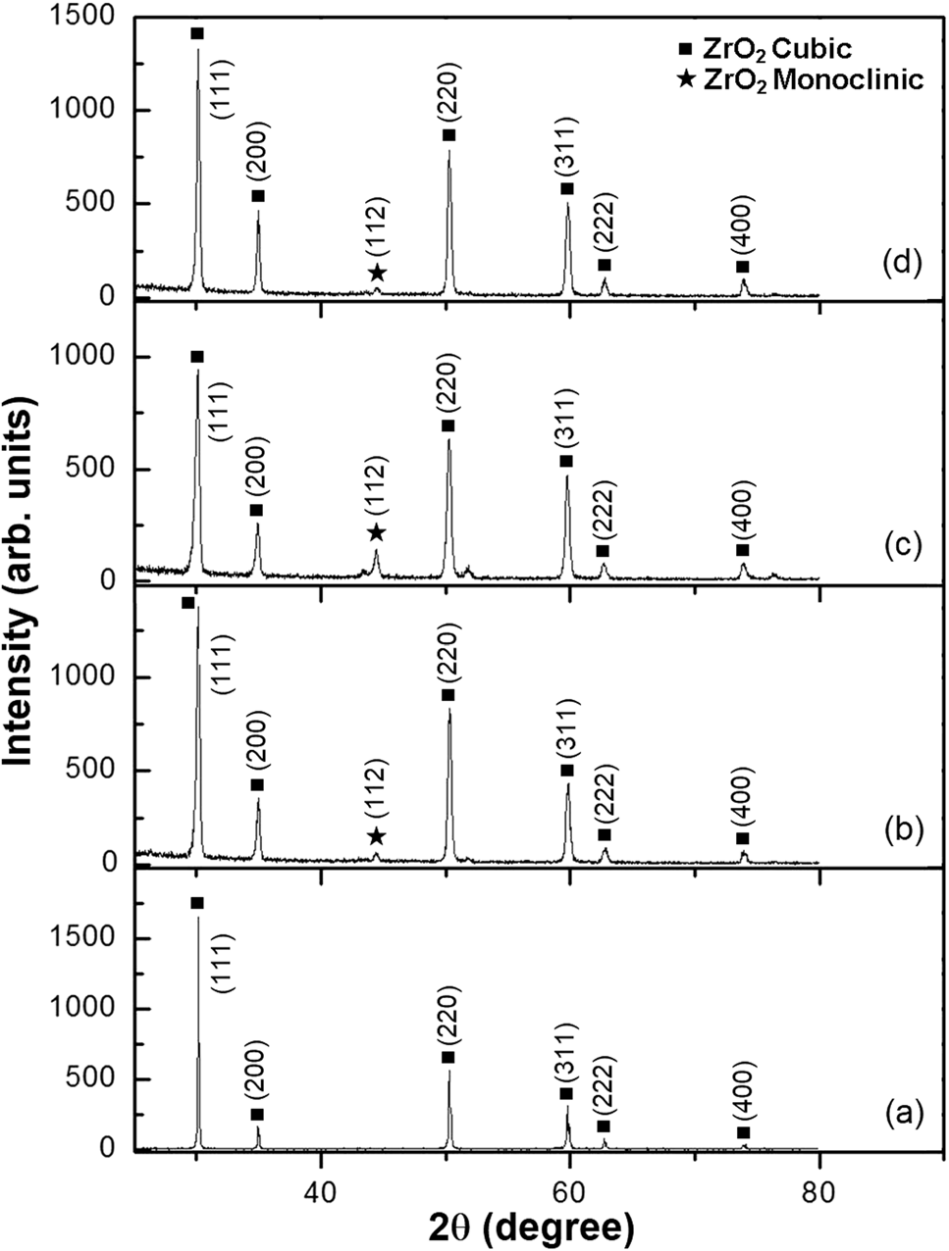
XRD patterns of the as-sprayed CSZ coating in (**b**), (**c**), and (**d**) with plasma spraying input currents compared with the CSZ powder in (**a**). Coatings in (**b**), (**c**), and (**d**) were corresponding to samples ZC1, ZC2, and ZC3, which were carried out at plasma spraying current of 600, 650, and 700 A, respectively.

**Figure 2:**
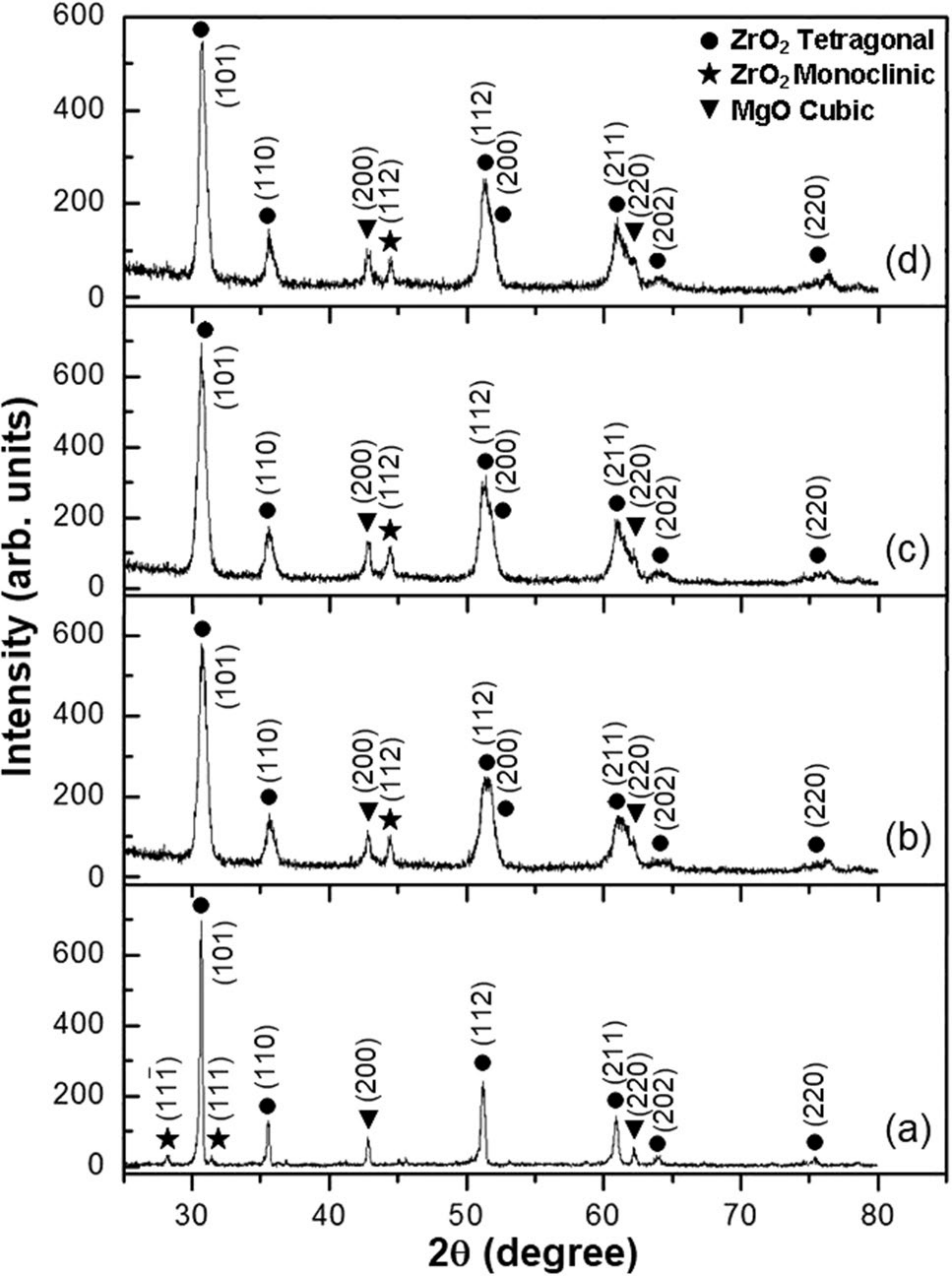
XRD patterns of the as-sprayed MSZ coating in (**b**), (**c**), and (**d**) with plasma spraying input currents compared with the MSZ powder in (**a**). Coatings in (**b**), (**c**), and (**d**) were corresponding to samples ZM1, ZM2, and ZM3, which were carried out at plasma spraying current of 600, 650, and 700 A, respectively.

XRD diffraction patterns showed the phase constituents of the as-sprayed three coating systems. XRD patterns of the CSZ powder, Fig. 1a, and top coatings of ZC1, ZC2, and ZC3 samples versus input different currents for plasma spraying, Fig. 1b–d, were compared. In case of the CSZ powder, the diffracted peaks of (111), (200), (220), (311), (222), and (400) are attributed to the zirconia cubic phase. The zirconia cubic phase of the as-sprayed top coatings was quite similar to the starting powder and there was no obvious degradation of crystallinity. XRD patterns of the MSZ powder, Fig. 2a, and top coatings of ZM1, ZM2, and ZM3 samples with different input currents for plasma spraying, Fig. 2b–d, were compared. Diffracted peaks such as (101) and (112) at 2θ = 30.7° and 51.2°, respectively, are characterizing the tetragonal phase. The cubic MgO phase emerged with peaks of (200) and (220) observed at 2θ = 42.8° and 62.2°, respectively. Weak traces of the monoclinic phase around 2θ = 28.2° and 31.4° for (111) and ($\bar{1}$11) diffraction peaks, respectively, were found in the initial MSZ powder. In addition, broadening of dominant tetragonal lines can clearly be observed for the (101) and (110) diffraction peaks. It can be seen that peak overlap occurred between (112) and (200) of tetragonal zirconia, and additionally between (211) and (220) of tetragonal zirconia and cubic MgO phases, respectively. XRD patterns of the as-sprayed CSZ and MSZ coatings samples revealed the presence of (112) diffraction peak around 2θ = 44.4° indicating the formation of a small amount of the monoclinic phase. The cubic and tetragonal phases were present in the initial zirconia powders and retained in the APS coatings of CSZ and MSZ.

**Figure 3:**
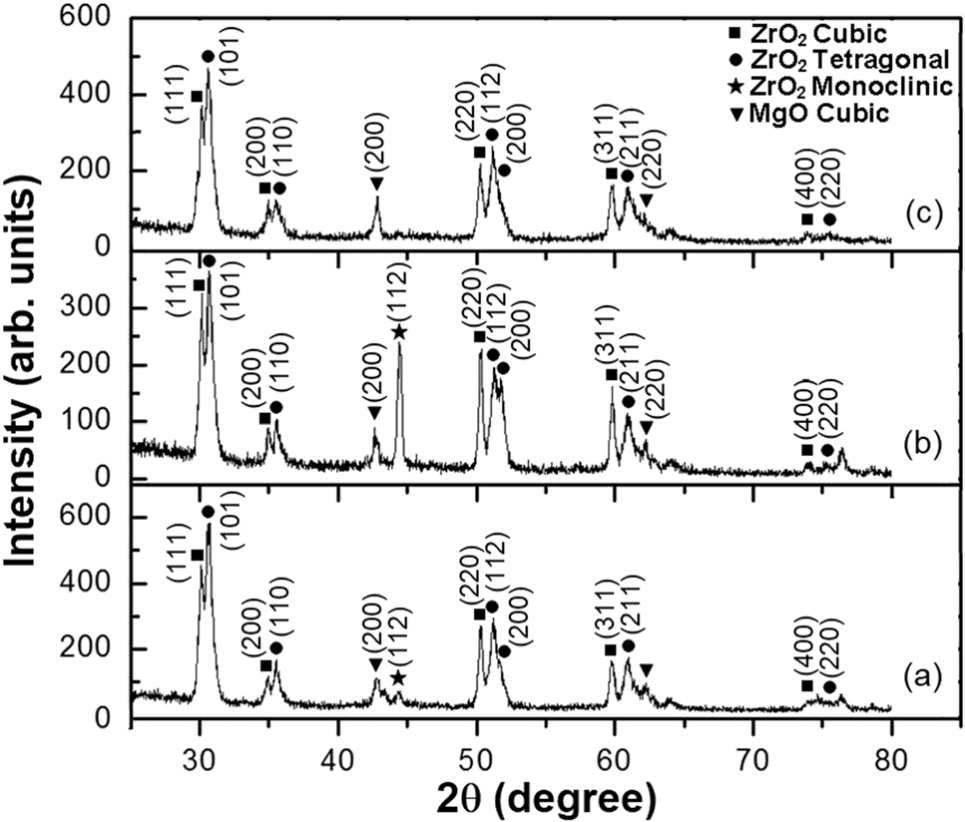
XRD patterns of the as-sprayed mixed CSZ-MSZ coatings with plasma spraying input currents. Coatings in (**a**), (**b**), and (**c**) were corresponding to samples Mix1, Mix2, and Mix3, which were carried out at plasma spraying current of 600, 650, and 700 A, respectively.

Figure 3 shows the phase composition of the as-sprayed mixed CSZ-MSZ coatings obtained with different input currents for plasma spraying. The as-sprayed coatings Mix1, Mix2, and Mix3 consist of a mixture of cubic and tetragonal phases. While there are small angular differences between the diffracted peaks, the cubic and tetragonal zirconia can be distinguished from the XRD patterns in Fig. 3. It is obvious that grown CSZ and MSZ grains were well aligned in the (111) and (101) orientations, respectively. Quantitative analysis of polymorphic mixes of zirconia by XRD was reported to identify the cubic and tetragonal phases from the low-angle {200} diffraction lines^38^. In order to overcome the difficulty of coincident diffraction lines, evaluation of high-angle diffraction data (72° ≤ 2θ ≤ 75°) was proposed^21,39^. The (400) cubic diffracted peak in this angle region may be utilized for phase analysis. As seen in Fig. 1, the (400) diffracted peak was observed for ZC1, ZC2, and ZC3 samples at 2θ = 73.9°. As well, the (400) peak was appeared in the mixed zirconia coatings, Fig. 3. No change in cubic and tetragonal zirconia phases was observed except that concentration of monoclinic phase increased obviously for the Mix2 sample. This implied that partial phase transformation from the cubic and tetragonal phases to the monoclinic phase occurred. While the diffraction peak intensity of (112) was mostly suppressed in the case of the as-sprayed Mix3 sample, suggesting a decrease in the monoclinic phase.

### Microstructure and elemental composition characterizations

**Figure 4:**
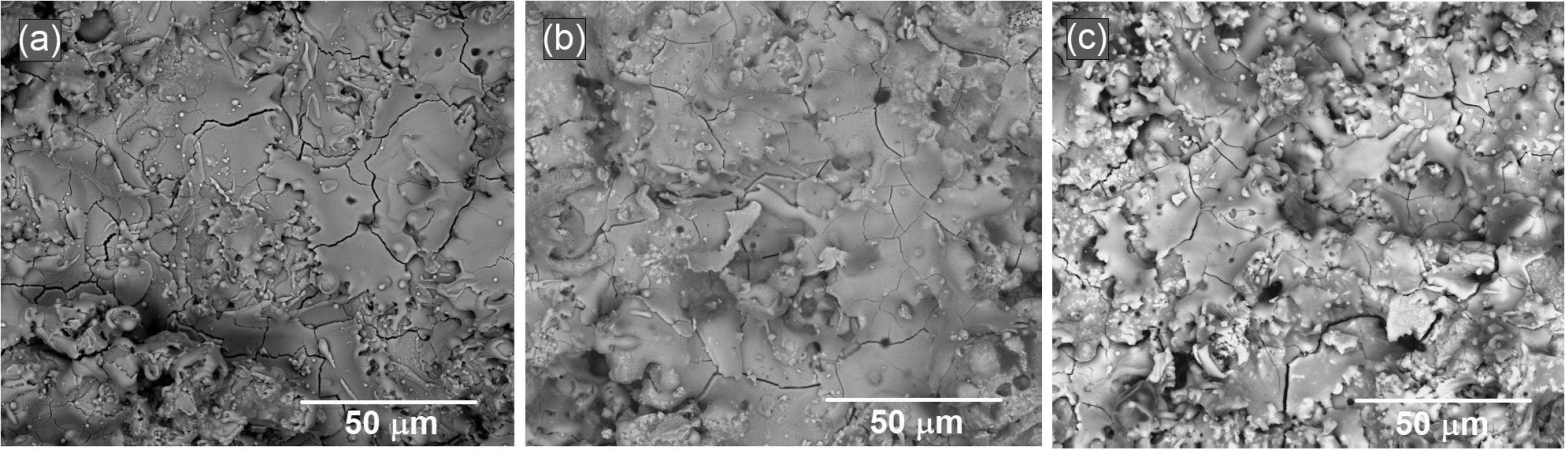
SEM images of as-sprayed top coatings CSZ, MSZ, and CSZ-MSZ by the APS showing surface microstructure of ZC3, ZM3, and Mix3 samples in (**a**), (**b**), and (**c**), respectively. Coated were performed on the 304 stainless steel substrate at plasma current of 700 A.

**Figure 5:**
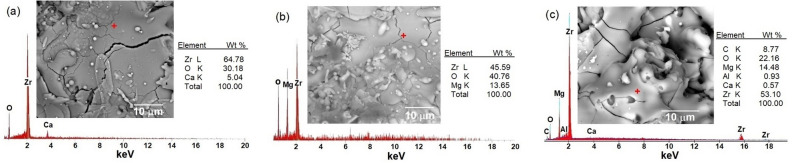
EDX spectra of the as-sprayed top coatings showing elements composition: (**a**) ZC3, (**b**) ZM3, and (**c**) Mix3. SEM images and table of elements (in wt%) are shown for the examined samples. The spots where EDX were performed are represented as cross mark on the corresponding SEM images. Coatings were performed on the 304 stainless steel substrate at plasma current of 700 A.

Observations of surface microstructure were made by the SEM on as-sprayed top coatings CSZ, MSZ, and CSZ-MSZ. Samples ZC3, ZM3, and Mix3 in Fig. 4 shows predominantly rough surfaces of the top coatings because of the speed at which particles arrive and the fast cooling rate associated with the APS method. The individual particles on surfaces may be partly melted or unmelted at the moment of impacting the substrate. The surfaces of the coatings showed microcracks and pinholes. Such features are typically occurring in these types of coatings by the APS. Top coatings have signs of impinging droplets of coating material. Deformation of molten particles by impacting the surface led to lateral spreading, which quenched and solidified forming splats. The observed crack patterns as seen in Fig. 4 were caused by residual stresses introduced at coating deposition. Unmelted or resolidified particles that trapped in the coating are common sources of coating porosity, which firmly influence coating properties. Figure 5 shows EDX spectra and SEM images of the as-sprayed coated samples. The composition of the EDX shows that the coated samples contain Zr, Ca, and Mg. The composition of the base material was maintained in the plasma spraying of the mixed CSZ-MSZ powders. EDX analysis indicated that ZM3 coating layers have oxides content of ~ 40% (wt%) higher than that of ZC3 and Mix3 coating layers, which have ~ 30% and ~ 22%, respectively.

**Figure 6:**
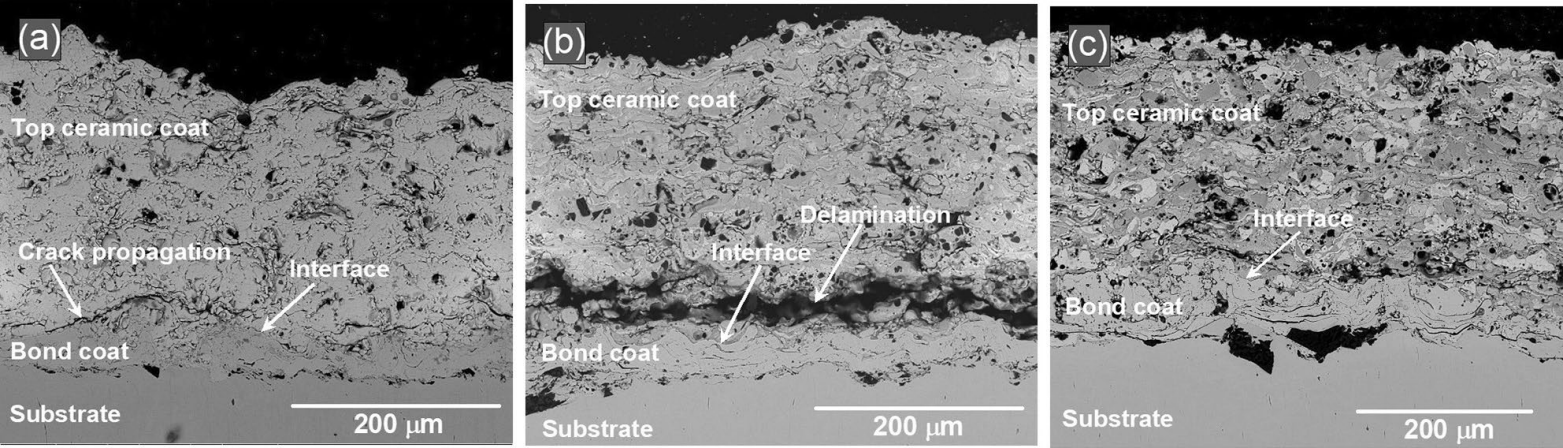
Cross-sectional SEM images showing crack propagation and coating delamination of as-sprayed top coatings in (**a**) CSZ and (**b**) MSZ, respectively. (**c**) Shows adherent interface between the Ni–5Al bond coat and the top ceramic CSZ-MSZ mixed coating. Coated samples of ZC3, ZM3, and Mix3 in (**a**), (**b**), and (**c**), respectively, were performed on the 304 stainless steel substrate at plasma current of 700 A.

Cross-sectional morphology and interfaces between dissimilar materials are the locations of interest because of the mismatch in their properties, such as elastic modulus and coefficient of thermal expansion (CTE)^40^. Comparison of cross-sectional SEM images of the three as-sprayed coatings is shown in Fig. 6. Obviously the bond layer can be clearly distinguished from top coatings. Individual splats can be clearly identified in these figures which revealed the characteristic lamellar structures of the thermal spray coatings. Overlapped splats were solidified and adhered to each other, creating continuous layers of coating. The splat structure of the deposited coating is indicative of the degree of melting of particles achieved prior to impacting the surface. The coatings show different levels of pores, which depend on the thermal spraying process and the spraying materials. Figure 6a of the CSZ coating revealed a progressive crack along the interface of the top coating and the Ni-5Al bond coating. Mismatch in the CTE of the coating components induced residual stresses, which act on a large scale leading to the cracking of the coating. In addition to the effect of differences in the CTE, the oxide content in the sprayed coating may have a major role in affecting the bond strength. It can be seen in the cross-sectional SEM in Fig. 6b the apparent delamination occurred between the top coat and the Ni-5Al layer. Because of increased residual stresses at the MSZ/Ni-5Al interface, crack growth reached a critical size that led to top coat delamination. EDX analysis conducted at the bond coat showed a large amount of Ni for the three coating systems. When Ni is oxidized, the formed oxide may grow within the ceramic coating and result in TBC delamination during extended air thermal exposure^20^. Traces of Fe and Cr were detected at the bond coat for the ZC and ZM samples. At high temperature exposure, the elements Fe and Cr from the substrate were diffused out to the bond layer. As well, a small amount of Zr was detected in the bond coat in the three coating systems. EDX analysis of the delaminated region for the ZM sample showed a high Al concentration, indicating preferential growth of Al-rich oxide near the bond coat/top coat interface. Open porosity and cracks can enable oxidizing elements to attack the base material^14^. The EDX analysis indicates the formation of rich Al_2_O_3_ and NiO, which could contribute to the increase of the interface stresses and to the delamination the MSZ layer. On the other hand, the mixed CSZ-MSZ coating sample exhibited an adherent interface with the bond layer as seen in Fig. 6c.

**Figure 7:**
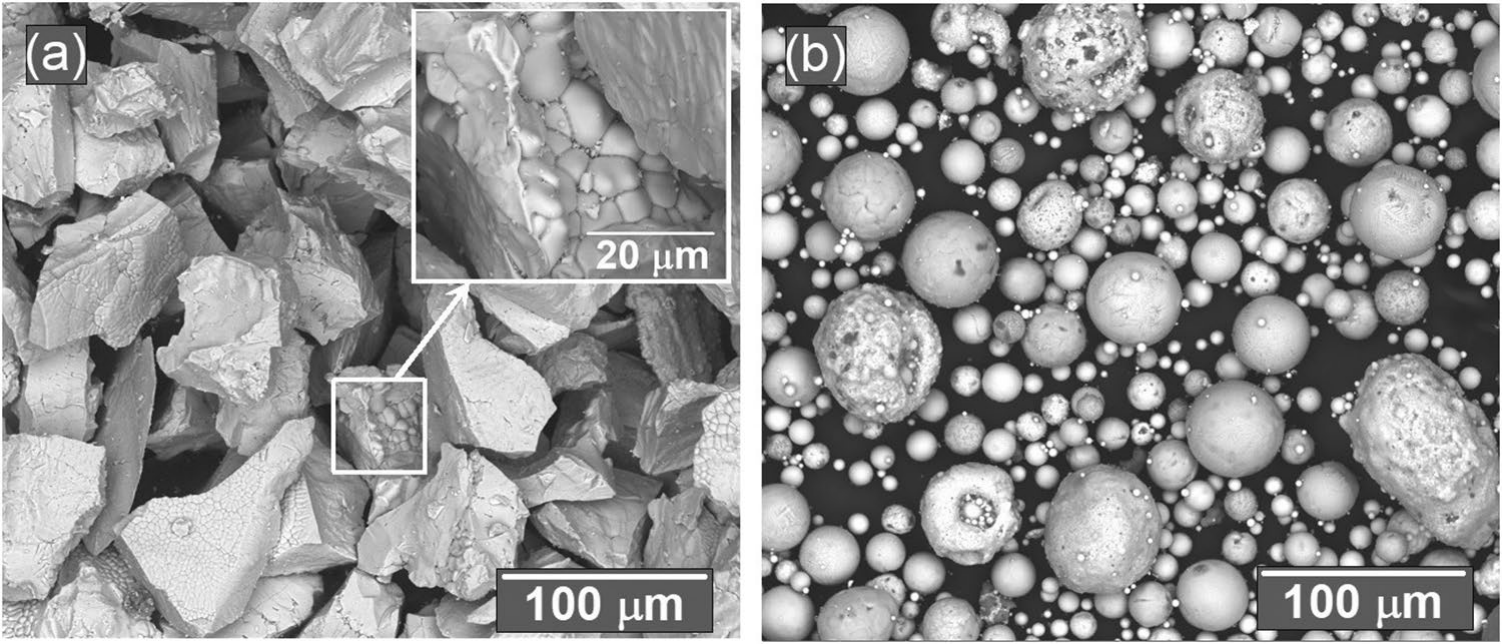
SEM images showing morphology of the as-received CSZ and MSZ feedstock powders in (**a**) and (**b**), respectively. The in situ in (**a**) is a high magnification SEM image of CSZ granulated particle showing micrograins feature.

**Figure 8:**
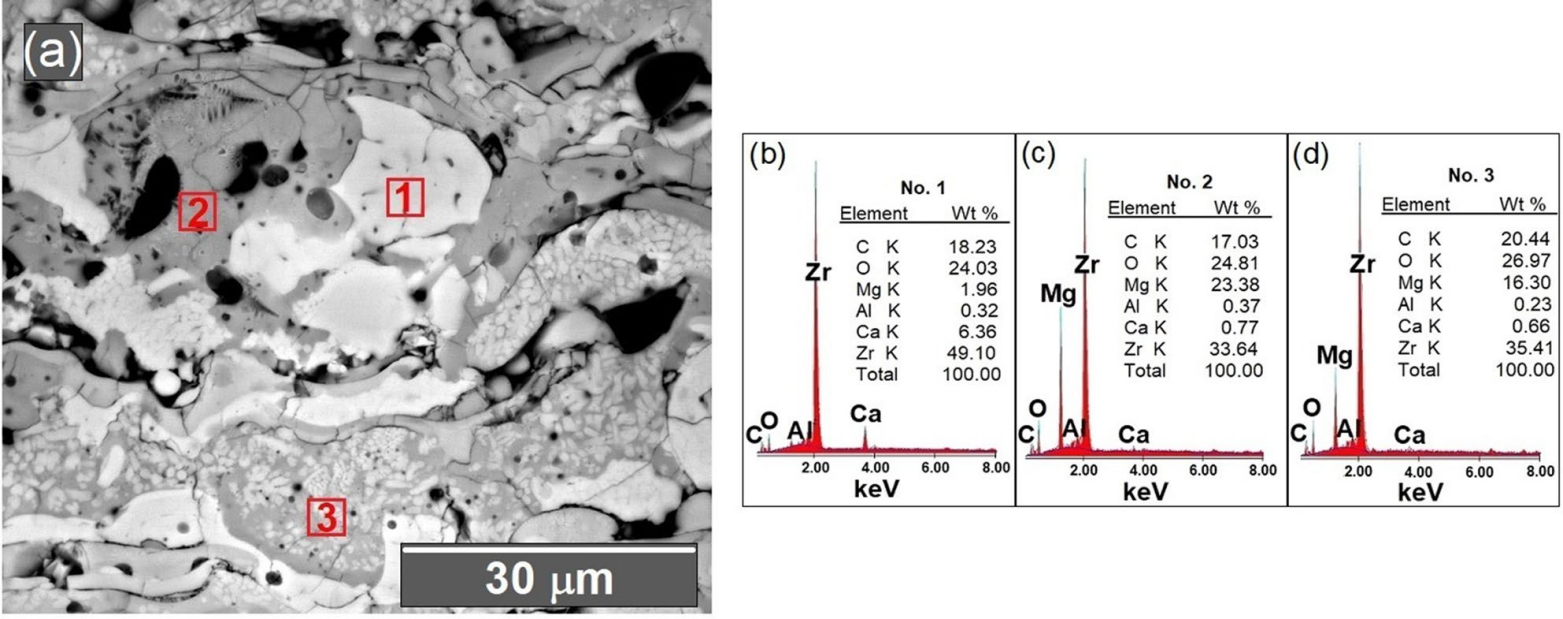
(**a**) Cross-sectional SEM showing microstructure of the as-sprayed mixed CSZ-MSZ top coating. Coating was performed on the substrate at plasma current of 700 A. EDX spectra in (**b**), (**c**), and (**d**) has taken on point mode at three spots marked as 1, 2, and 3 within three different regions of the coating as depicted, which shows the differences in elemental compositions in these regions.

Throughout spraying, the morphology of the CSZ-MSZ coating is correlated to the melting degree of the mixed powders and the distribution of injected particles within the plasma jet. The overall morphology of the as-received CSZ and MSZ feedstock powders were examined by the SEM. Figure 7a,b display SEM images of angular/blocky and spheroidal feeding particles of the CSZ and MSZ powders, respectively. High magnification SEM image shows that CSZ particles comprised of micrograins. Figure 8a showed cross-sectional SEM taken for the top ceramic coat of the Mix3 sample. It is seen that the top layer is comprised of different regions of white, gray and gray-white mixture, which can be distinguished by the diversity of the microstructure contrast. Elemental composition by EDX at selected sites of the white, gray and gray-white mixture regions are shown in Fig. 8b–d. The EDX analysis corresponds to those regions showed Zr, Ca and Mg with different contents (in wt%). EDX indicated that Zr ranged from 33 to 49%. The white region was rich in Ca (~ 6%) whereas the grey region was rich in Mg (~ 23%). While the presence of C peak on the EDX analysis is caused by the carbon layer, which was used for coating the surface of the ceramic coating/layer. At the gray-white mixture region, the SEM image showed white fine particles dispersed in grey grain. In this region, EDX pattern indicated a composition of 16:0.6 wt% Mg:Ca. These variations in the microstructure of the coating were dependent on the thermal conditions within the APS process and the characteristics of the injected particles. It is inferred that the melted CSZ and MSZ particles were mixed to form microsized ceramic-matrix grains with different compositions. The possibility that fine CSZ particles were embedded in MSZ grain was thought to be due to incomplete melting prior to the deposition on the surface of the substrate. The coating microstructure shows that fine CSZ particles were dispersed within the MSZ matrix grains forming intragranular structure, and furthermore occupied grain boundaries forming intergranular structure, Fig. 8a.

### Mechanical properties

**Figure 9:**
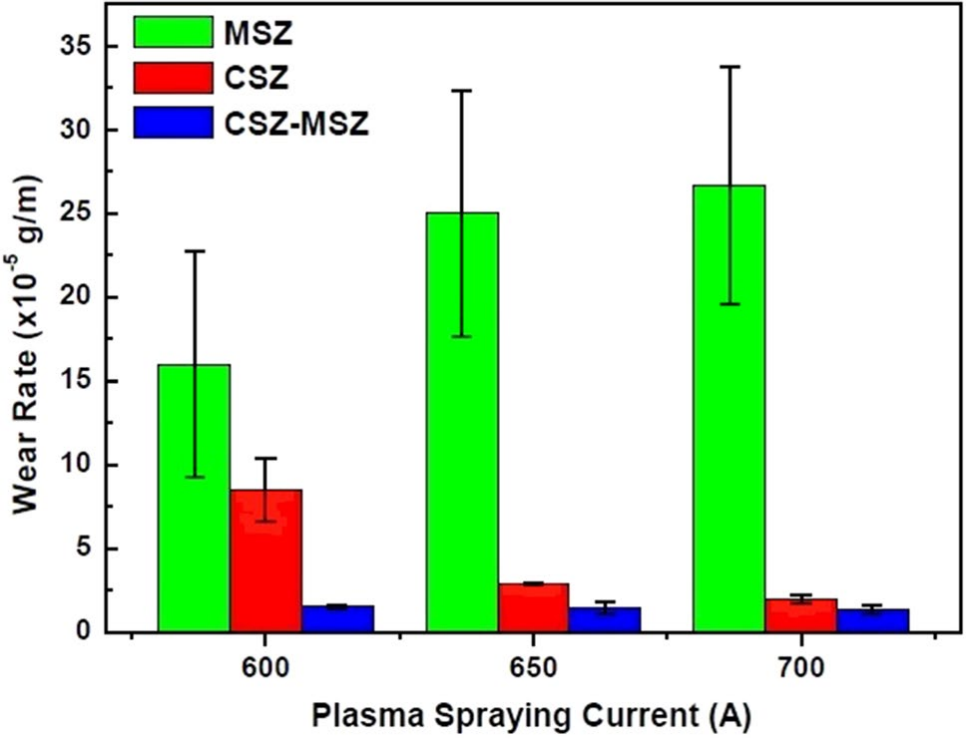
Wear rates of CSZ, MSZ, and CSZ-MSZ coatings were compared at coating thicknesses of 300 μm at plasma current of 600, 650, and 700 A. Coated samples of the CSZ, MSZ, and CSZ-MSZ powders with different plasma spray conditions were designated as ZC, ZM, and Mix, respectively. Error bars represent the standard deviation.

The as-sprayed coated samples with different spraying conditions were subjected to the abrasive wear test using the pin-on-ring process. The wear rates were measured from the cumulative weight loss of the coating against sliding distance. After deposition of the initial coating layer and as the thickness increases, sprayed particles impact on a much harder surface (the previously deposited coating layers)^41^. The melted particles and droplets will flatten more efficiently on next coating layers and fill underlying interstices. This results in a reduction of the voids and around surface irregularities. As the underlying layers were densified by the deposition, the coating became less defective. The wear rates for CSZ, MSZ, and CSZ-MSZ coatings at 300 μm thickness were compared with the plasma spraying input currents, as seen in Fig. 9. For example, at a current of 700 A, the wear rate of the Mix3 sample was about 1.4 × 10^–5^ g/m compared to approximately 2 × 10^–5^ and 26.7 × 10^–5^ g/m of the ZC3 and ZM3 samples, respectively. The wear resistance of the mixed CSZ-MSZ coating showed obvious improvement followed by CSZ coating. In contrast, the MSZ layer showed the highest wear rate, which was increased by increasing the current amperes.

**Figure 10:**
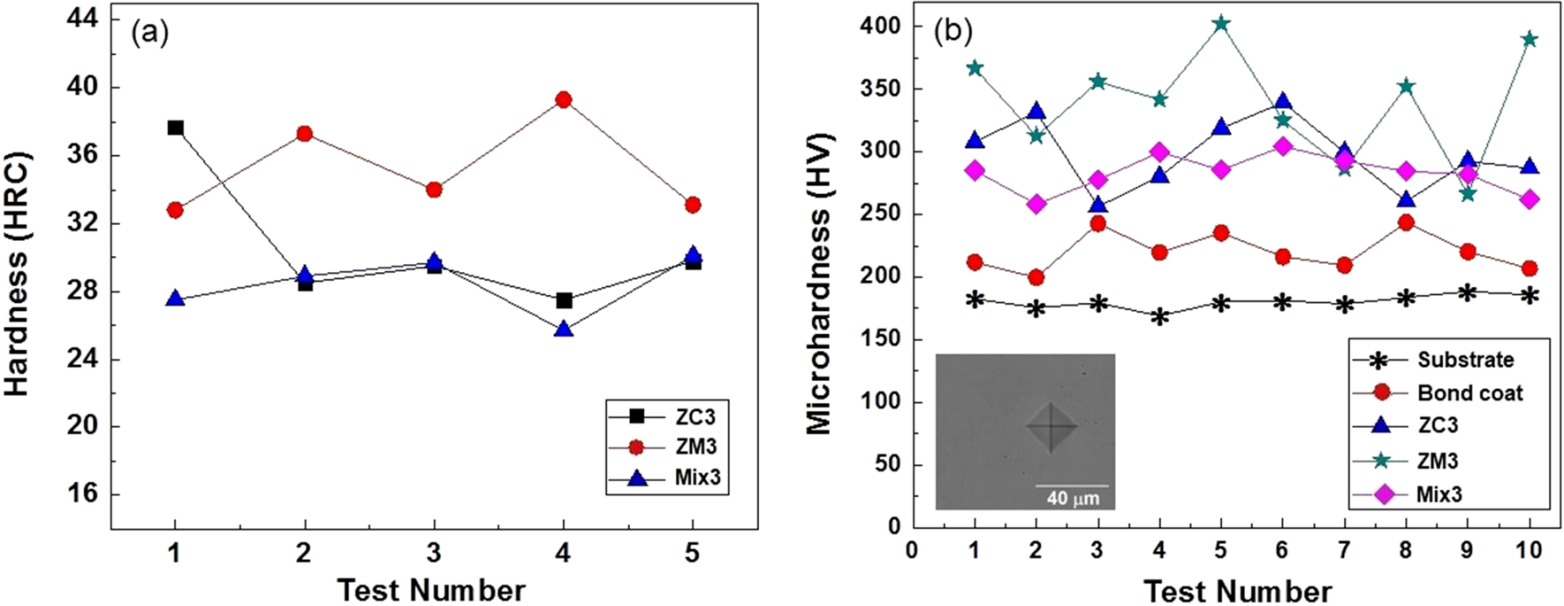
(**a**) Rockwell hardness carried out on the surfaces of the ZC3, ZM3, and Mix3 samples coated at 300 μm thickness and plasma spraying current of 700 A. (**b**) Vickers microhardness variation on the cross-section of the substrate, bond coat, and top coats of ZC3, ZM3, and Mix3 samples (ten indentation per sample) coated at 300 μm thickness and plasma current of 700 A. The inset is a SEM image show the Vickers indent on the cross-section of the substrate under a 500-g load.

Rockwell hardness was carried out on the surfaces of the samples that coated at a thickness of 300 μm and a plasma current of 700 A (Fig. 10a). Sample ZM3 showed relatively higher surface hardness than the ZC3 and Mix3 samples. The depth of the indentation is proportional to the surface layers conditions, and essentially the average hardness of many individual grains. Vickers microhardness was performed in the cross-section of the 300 μm thick top coated samples (ten indentations per sample) and a plasma current of 700 A. measurements were made approximately in the middle of the thickness of the coatings. Variations in the microhardness of the substrate, bond coat, and top coats were compared in Fig. 10b. The typical SEM image of the Vickers indent taken from the cross-section of the substrate is shown in Fig. 10b. On average, the microhardness of ZM3 coating was 339 HV_0.05_, while the ZC3 and Mix3 coatings were shown to be 297 and 283 HV_0.05_, respectively. The magnitude of the microhardness is strongly influenced by the variety of microstructure defects. The distribution of the microhardness of the mixed CSZ-MSZ layer showed a better higher level of consistency than the individual CSZ and MSZ layers.

**Figure 11:**
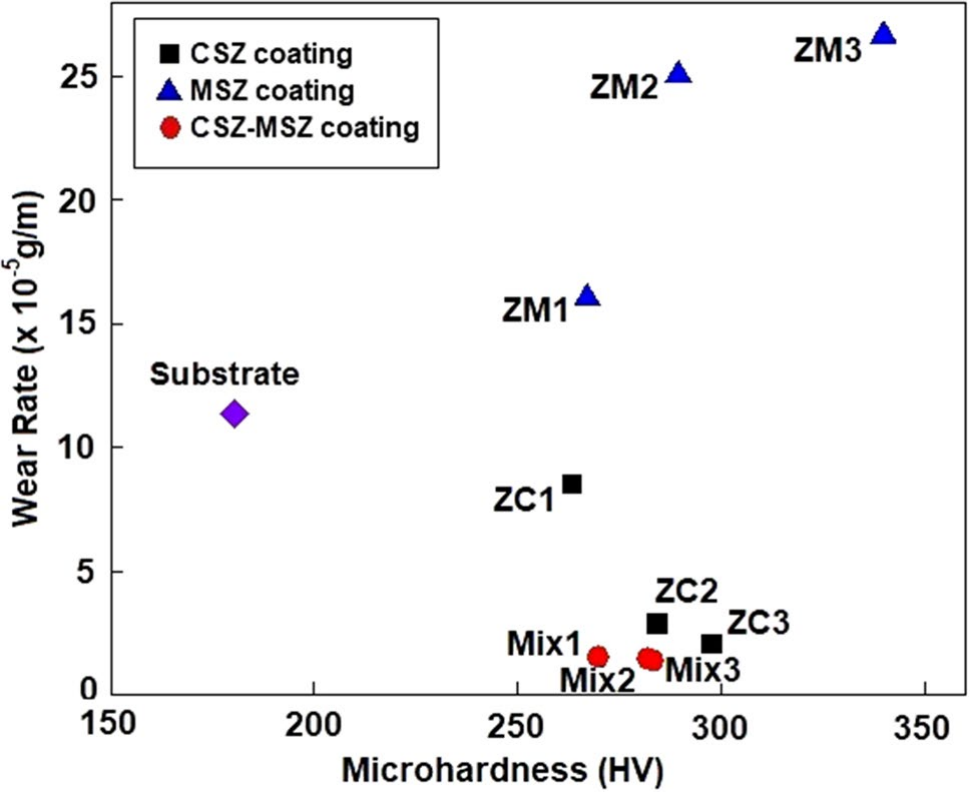
Relationship between the measured wear resistance and microhardness of CSZ, MSZ, and CSZ-MSZ coatings at thickness of 300 μm. The substrate microhardness and wear resistance is provided for reference.

The relationship between the wear resistance and the microhardness of the zirconia coatings at a thickness of 300 μm is shown in Fig. 11. The highest microhardness of 339 HV_0.05_ characterizes the ZM3 sample, while the lowest wear rate of approximately 1.4 × 10^–5^ g/m characterizes the Mix3 sample. According to Fig. 11, there is a direct correlation between the wear resistance and the microhardness of the CSZ and CSZ-MSZ coatings. However, the MSZ coating showed different wear resistance behavior, which is the higher the microhardness, the lower the wear resistance. These results indicate the importance of the characterization of the injected powders and its effect on the internal microstructure of the as-deposited zirconia coatings by the APS.

## Discussion

The results showed that potential improvement in wear resistance was achieved by plasma spraying of mixed calcia and magnesia stabilized-zirconia powders. The top coating of the mixed CSZ-MSZ was well bonded to the Ni-5Al bond coating and has superior wear resistance compared to the individual CSZ and MSZ coatings. The effects of phase stability, microstructure development, and associated residual stresses on wear resistance of the mixed CSZ-MSZ coating are investigated.

The degree of phase stability of the zirconia coating depends on the stabilizer content, phase composition of the initial powder, and temperature exposure. In the present work, occurrence of metastable phases of zirconia was ruled out on the basis of the absence of characteristic peaks in XRD patterns of the as-sprayed samples. The XRD phase analysis showed that the three APS coating systems of ZrO_2_ composed of cubic and tetragonal phases. It can be seen that the cubic and tetragonal structures were stable under plasma spray conditions. However, the XRD patterns of the ZC2, ZM2, and Mix2 samples, Figs. 1c, 2c, and 3b, respectively, showed a fluctuating proportion of (112) monoclinic peak at 2θ = 44.4°. In comparison, the XRD of the ZC3 and Mix3 samples indicated that monoclinic zirconia phase had been suppressed. The amount of the monoclinic phase of as-sprayed ZrO_2_ coating is one of the important measures of coating quality. Diffracted lines broadening in the XRD of the CSZ and MSZ coatings, relative to their sharpening in case of the starting powders, were related to grain size. From the viewpoint of the grain size effect, creation of small crystallites at high temperature exposure gives rise to line broadening of their XRD patterns. Transformation into the monoclinic phase of the plasma-sprayed stabilized ZrO_2_ coatings was found to be strongly dependent on grain size^24^.

The resultant XRD patterns of mixed coating were relatively similar to the individual CSZ and MSZ coatings. Irrespective of heat exposure, the retention of cubic and tetragonal phases is thought to be due to a contribution from two reasons. First, the CaO and MgO were successfully doped in the zirconia lattice and preserved the cubic and tetragonal phases at ambient temperature. Secondly, rapid cooling of plasma spraying was effective in suppressing phase transformation to the monoclinic phase, such as in the coated samples of ZC3 and Mix3 (Figs. 1d, 3c). The molten particles cool rapidly, both during the flight to and after striking the substrate surface. The phase transformation of ZrO_2_ may occur during heating and cooling processes. On cooling, the low stabilizer tetragonal phase may transform to a monoclinic structure, which has a lower free energy at lower temperatures^39^. It has been thought that the stabilization of the tetragonal phase arises from a combination of the surface energy effect and the constraint of the rigid matrix that opposes the transformation to a less dense monoclinic form^42^. In a previous study, as-sprayed ZrO_2_–24MgO top coat showed a complete non-transformable tetragonal phase up to 750 °C, however, after thermal exposure at 1000 °C for 60 h, the transformation of the tetragonal phase to the monoclinic phase was observed in the XRD patterns^33^. Depending on the high temperature environment, the metastable non-transformable tetragonal can be expected to decompose into the cubic and tetragonal equilibrium phases by diffusion of the CaO and MgO stabilizers. However, the quenching process could prevent the compositional adjustment required for the development of equilibrium low-stabilizing transformable tetragonal phase^39^. Rapid cooling and solidification in the APS process restrained the diffusion of the stabilizing elements and therefore suppressed the nucleation and growth of the grains. This led to a restriction of grain sizes and prevented materials from achieving their equilibrium phases. Therefore, the diffusion of stabilizers and grain growth are competing with the rapid cooling which occurs during the spraying process, and which leading to control the phase transformation. From the XRD patterns, it can be concluded that the top coats retained high stabilizer non-equilibrium structures of non-transformable tetragonal or cubic phases, taking into account the rapid cooling and solidification.

The coating microstructure is correlated with the plasma spraying parameters and the deposition mechanism of the sprayed particles on the surface of the substrate. SEM cross-sectional and EDX analysis revealed modification of the top coating microstructure. This is shown in diversity of the as-sprayed CSZ and MSZ coating microstructures is shown in Figs. 6 and 8a. The growth of ceramic-matrix grains in the as-sprayed mixed CSZ-MSZ coating was influenced by the transport phenomena of powder particles by the APS process. It is possible that partially melted or solid particles and liquid droplets deposited simultaneously on the substrate surface may form dense regions of mixed CSZ and MSZ coating, as seen in Fig. 8a. Besides effects of the plasma jet process, the morphology and size distribution of the starting granules were also affecting the microstructure of TBCs^43,44^. Formation of different ceramic-matrix microstructure regions is attributed to differences in morphology and melting point between angular/blocky and spheroidal feeding particles of CSZ and MSZ, respectively. Upon impact the substrate surface, particles from all positions in the jet stream deposit virtually simultaneously^14^. As seen in Fig. 7, some CSZ granulated particles were comprised of micrograins, which indicating that grains packed together more weakly. This suggests that unmelted CSZ particles could be shattered on impact due to the high kinetic energy of the jet and the brittle nature of the powder. The resulting fine CSZ particles were embedded in the molten MSZ forming an intragranular microstructure, Fig. 8a.

Residual stresses become driving forces for crack propagation eventually causing the final failure of TBCs^27–29^. Therefore, investigation of failure mechanisms and their relation to residual stresses is an imperative aspect. The cooling generated in the zirconia coating after processing is superimposed with the ones introduced by crystal-phase transformation giving the residual stress. Moreover, under atmospheric conditions, oxidation is an important feature of the plasma spraying process as the oxides grow at the deposited layers and interfaces, which in turn affects the durability of the coatings. As a result, bond layer oxidation behavior in the APS process may lead to high stresses, which are favorable conditions for crack growth. As it is known the intermediate layer of NiAl is usually utilized as a primer metallic coating to improve the adhesion of the ceramic powders to the steel substrate. Therefore, it enhance the ceramic layer bonding in addition it also lowers the oxidation rate^45,46^. However, growth of the TGO layer on the bond coat during thermal exposure to air is accompanied by stress generation at the bond coat/top coat interface. The increase in TGO thickness may cause the delamination of the ceramic layer from the bond coat^47,48^. It was found that Al_2_O_3_ formed preferentially along the bond coat interface and other oxides such as NiO and Ni(Cr,Al)_2_O_4_ spinel formed rapidly at the top coat side of the interface at temperature above 1100 °C^20^. The growth of NiO and Ni(Cr,Al)_2_O_4_ spinels is more damaging because they have a very high growth rate which rapidly increases the volume of the bond coat^20^. It can be inferred that these oxide layers on the bond coat have increased due to the inward infiltration of oxygen and the outward diffusion of Al and Ni at high temperatures.

Transfer of oxygen through cracks and pores towards the bond coat plays an important role in the delamination of top coat^49,50^. In addition, growth of NiO and mixed oxides onto the TGO layer imposed excessive stresses on the zirconia layers and the MSZ/bond coat interface. The growth of these oxides at the bond coat/top coat interface increased the stress level. Relaxation of the residual stresses is correlated with nucleation and coalescence of cracks, thereby leading to delamination of the MSZ top coat. Another possible factor affecting the delamination of the top coat is the roughness and irregularities of the bond coat surface, which create preferred sites for stress concentration^51^. In designing APS of TBCs, suppressing oxide growth stress during high temperature thermal cycling is crucial to increase lifetime of the coating. Mixing CSZ and MSZ has the potential to play a role in improving the mechanical properties of zirconia coatings. The results indicated that the CSZ-MSZ mixed coating provided a diffusion barrier for oxygen infiltration than the individual CSZ and MSZ coatings. It is important to control the oxide content of APS zirconia coatings to an adequate level for a given application.

While mixed CSZ-MSZ coating showed the best wear resistance under plasma spraying conditions, CSZ and MSZ coatings showed different wear behaviors as seen in Fig. 9. Raising the plasma spray current improved the wear resistance of CSZ coating while in contrast it created more defects in the MSZ coating. The premise is that the higher the microhardness, the higher the wear resistance was not valid in the case of MSZ coating. This can be seen in the inverse correlation between the microhardness and the wear resistance of MSZ coating in Fig. 11. Understanding the growth of the coating and its related defects can provide insights into how microstructure coatings can be modified to minimize wear. In this study, the wear resistance was significantly influenced by the internal microstructure of the zirconia coatings. Sprayed deposits typically contain different levels of oxides, porosity, and unmelted particles, depending mainly on the characterization of the feedstock powders and the plasma spray parameters used. It has been reported that small particles produce splats that are bonded through oxide layers, while porosity increases with the particle size^52^. Besides the effects of particle size on the microstructure and the mechanical properties of plasma sprayed coating, other factors should be taken in consideration. First, the oxygen content in the initial powders of the calcia and magnesia stabilized zirconia possibly affect the size and density of the oxides in the coatings. Second, the homogeneity and crystalline phases of deposited coating have influenced the spatial distribution of defects. Disparity of these defects has a substantial impact on the mechanical properties of the coatings. The wear resistance of plasma sprayed zirconia could be more sensitive to a specific coating defect. It has been shown that the increase in Vickers microhardness of cermet coatings was accompanied by a significant decrease of the sliding wear rate^41^. Another study showed that the wear rates of plasma sprayed yttria stabilized zirconia coatings were linearly proportional to their microhardness^53^. On the other hand, it has been found that the wear resistance of the thermally sprayed WC-based coatings is not linearly proportional to the hardness^54–56^. Previous results of thermal spray tungsten and chromium carbides coatings indicated weekly dependence of wear resistance on the microhardness level^57^. The oxides and porosity are the most influential factors that cause a discrepancy effects on the mechanical properties of the thermal spray coatings. During high temperature exposure, thermally activated processes induce the growth of oxide in the coating layers. Excessive oxide network effects in the MSZ coatings can be realized from the EDX analysis as shown in Fig. 5 and the delamination of the top coat, as seen by SEM in Fig. 6. Our findings indicated that the MSZ coating had a relatively higher hardness than the CSZ and CSZ-MSZ coatings. These ultimately point out to the effects of the oxide content in the MSZ coating were opposite relationship was observed, where the hardness was high while the wear resistance was reduced. It can be inferred from the present results and the previous studies^41,53–56^ that the wear behavior of zirconia coatings is complicated^58^.

It should be mentioned that the conditions for the thermal spray process were kept constant for the samples (distance, voltage, current and feed rate) and therefore the porosity was expected to remain within same levels for the samples. This is further confirmed by the cross-sectional SEM observations as shown in Fig. 6 of a typical morphology of the coating for the powders. In our case, the typical porosity range in the coating of the CSZ and MSZ powders processed by APS and bond coated by NiAl layer are 5–10% and 5–8%, respectively (Metco 201B-NS and Metco 210NS-1 materials data sheets)^59^. Furthermore, particle morphology is an inherent source of controlling porosity in the coating^14^. Internal porosity of powders such as dimpled and hollowed particles was found to affect the final properties of coating^60^. In our study, SEM images in Fig. 7 showed dense microstructure of the feeding powders, which were angular/blocky and spheroidal of the CSZ and MSZ particles, respectively. Under these conditions of thermal spray, porosity is essentially determined by the average particle temperature distribution and velocity distribution. With approximately the same levels of porosity, the wear resistance of the studied samples was mainly dependent on the intersplat cohesive bonds and in turn the oxide content.

The wear resistance of the plasma sprayed coatings is correlated with the lamellar structure, which is built up of splat-on-splat structure. It has been reported that wear mechanisms of plasma sprayed yttria stabilized zirconia coatings were attributed to adhesion-induced spallation and micro-fracturing of lamellae^53^. The intersplat cohesive strength has been shown to be the principle factor affecting wear performance^61^. Accordingly, wear resistance indicates how the deposited splats on the substrate are well-interrelated. The high weight losses of the MSZ coatings, due to abrasive wear, were related to weakly intersplat cohesive strengths. While the wear rate of CSZ coating was improved from ~ 8.5 × 10^–5^ to ~ 2 × 10^–5^ g/m, MSZ coating suffered excessive wear debris from ~ 16 × 10^–5^ to ~ 26 × 10^–5^ g/m (Fig. 9). Although the CSZ powder has a low oxide content, its downward trend of the wear rate was different relative to the mixed CSZ-MSZ coating (Fig. 9). The oxides are generally concentrated on the splat surfaces and aggregated around the splat boundaries^52^. Therefore, improving the wear resistance of zirconia coating could be realized by increasing the intersplat cohesive bonds. The presence of coating oxides would be under control if the growth of ceramic-matrix is developed for the required material strength. This was achieved in this work by plasma sprayed mixed powders of CSZ and MSZ, which resulted in a modification of the coating microstructure, as seen in the SEM image of Fig. 8a. The wear mechanism was affected by the microstructural heterogeneities such as grain boundaries, porosity, and microstructure. High performance wear resistance could be achieved when sprayed particles are reconstituted both in grains and along the grain boundaries of the coating to form intragranular and intergranular microstructures (Fig. 8a). These microsized matrix grains in the case of mixed CSZ-MSZ coating proceeded as strong barrier to diminish growth and diffusion of oxides in the vicinity of the coating, which increased the intersplat cohesive strength. One benefit accomplished was the overcoming of the delamination between the top coat and bond coat. Furthermore, the wear resistance of mixed CSZ-MSZ coating has potentially improved compared to the individual calcia and magnesia coatings. It should be emphasized that the wear rates of the mixed CSZ-MSZ coatings reduced dramatically with a value of 1.4 × 10^–5^ g/m with the applied plasma input currents (Fig. 9). This further suggests that plasma spraying of the mixed CSZ-MSZ coating could be processed at lower temperatures to attain benefit in limiting in-flight oxidation of sprayed particles, which could lead to efficient contact between the splats.

## Conclusions

Thermal spray of mixed calcia and magnesia stabilized-zirconia (CSZ-MSZ) powders found to be an effective approach to enhance the wear resistance of the obtained coating. The as-sprayed CSZ-MSZ coating consists of both cubic and tetragonal phases that stabilized under different APS spray conditions. Growth of modified ceramic-matrix grains was influenced by the initial characteristics of the powders and by APS process parameters (power level). Investigation of the resultant mixed coating emphasized that wear resistance and microhardness of the coating depend on the observation of growth of oxide-matrix microstructures. This growth increased the intersplat cohesive strength and prevented delamination. In the case of mixed CSZ-MSZ coating, the wear resistance significantly improved in comparison to either individual CSZ or MSZ coatings. Better understanding of oxide growth and microstructural process of APS in mixed CSZ-MSZ coating will allow an improvement in mechanical properties and prolongation of the TBC lifetimes.

## Data Availability

The data generated and analysed during the current study will be made available from the corresponding author on reasonable request.
